# Fusion of Mid-Wave Infrared and Long-Wave Infrared Reflectance Spectra for Quantitative Analysis of Minerals

**DOI:** 10.3390/s20051472

**Published:** 2020-03-07

**Authors:** Feven Desta, Mike Buxton, Jeroen Jansen

**Affiliations:** 1 Resource Engineering Section, Department of Geoscience and Engineering, Delft University of Technology, Stevinweg 1, 2628 CN Delft, The Netherlands; m.w.n.buxton@tudelft.nl; 2 Department of Analytical Chemistry: Chemometrics, Faculty of Science, Radboud University, P.O. Box 9010, 6500 GL Nijmegen, The Netherlands; jj.jansen@science.ru.nl

**Keywords:** MWIR, LWIR, polymetallic sulphide ore, minerals, data fusion, PLSR, PCR, SVM

## Abstract

Accurate quantitative mineralogical data has significant implications in mining operations. However, quantitative analysis of minerals is challenging for most of the sensor outputs. Thus, it requires advances in data analytics. In this work, data fusion approaches for integrating datasets pertaining to the mid-wave infrared (MWIR) and long-wave infrared (LWIR) spectral regions are proposed, aiming to facilitate more accurate prediction of SiO_2_, Al_2_O_3_, and Fe_2_O_3_ concentrations in a polymetallic sulphide deposit. Two approaches of low-level data fusion were applied to these datasets. In the first approach, the pre-processed blocks of MWIR and LWIR data were concatenated to form a fused data block. In the second approach, a prior variable selection was performed to extract the most important features from the MWIR and LWIR datasets. The extracted informative features were subsequently concatenated to form a new fused data block. Next, prediction models that link the mineralogical concentrations with the infrared reflectance spectra were developed using partial-least squares regression (PLSR), principal component regression (PCR) and support vector regression (SVR) analytical techniques. These models were applied to the fused data blocks as well as the individual (MWIR and LWIR) data blocks. The obtained results indicate that SiO_2_, Al_2_O_3_, and Fe_2_O_3_ mineral concentrations can be successfully predicted using both MWIR and LWIR spectra individually, but the prediction performance greatly improved with data fusion; where the PLSR, PCR, and SVR models provided good and acceptable results. The proposed approach could be extended for online analysis of mineral concentrations in different deposit types. Thus, it would be highly beneficial in mining operations, where indications of mineralogical concentrations can have significant financial implications.

## 1. Introduction

In mining, access to accurate quantitative mineralogical data has significant implications for the production process efficiency. Quantitative mineralogical data, available in real-time, would greatly assist material characterization (e.g., ore versus waste), operational decision making, optimization of ore processing, and the specification of product quality. Understanding material composition in mineral processing can also minimise the technical and financial risks. It may thereby greatly enhance economic, safety, and environmental performance of a mining operation.

Geometallurgical investigation links the geological and mineralogical characteristics to the metallurgical performance of an orebody. It is an important approach to optimize resource efficiency and reduce the technical risk associated with mining operations. The required information for geometallurgical applications is not limited to knowledge on the grades of valuable elements and their variability, but also extends to the gangue minerals, as their composition and volume also play a crucial role in ore processing. Extant studies highlight the importance of mineralogical information for the sustainability and energy efficiency of geometallurgical processes [1,2]. Ore minerals occur in veins, disseminated in the host rock and/or in pores with varying concentrations of other associated minerals such as silica, oxides and carbonates. The concentration of these minerals can be associated with the metallurgical behaviour of the ore minerals. Therefore, quantitative mineralogical information on the co-occurring minerals is one of the crucial parameters for the optimisation of ore processing. 

Despite rapid advances in sensor technologies, there is still a demand for novel ideas to enable quantitative investigations of mineralogical compositions using sensor-derived data. In addition, in-situ application of sensor technologies requires portable and high-speed systems. Portable sensor technologies (such as X-ray fluorescence—XRF, and short-wave infrared—SWIR) that provide geochemical or mineralogical data are available. However, most of the currently available sensor technologies are laboratory-based techniques. Owing to the growing interest in an accurate, in-situ and on-line quantitative analysis of minerals, infrared technologies coupled with advanced data analytics can be promising alternative tools. Despite rapid advances in sensor technologies, there is still a demand for novel ideas to enable quantitative investigations of mineralogical compositions using sensor-derived data. In addition, most of the currently available sensor technologies are laboratory-based techniques. Owing to the growing interest in an accurate, in-situ and on-line quantitative analysis of minerals, infrared technologies coupled with advanced data analytics can be promising alternative tools. Infrared (IR) spectroscopy is a well-established analytical technique that can be applied in qualitative and quantitative analysis of organic and inorganic materials [3,4,5]. State-of-the-art infrared technologies are fast, portable, non-destructive, and can operate over a wide electromagnetic spectral range [6,7]. The IR region of the electromagnetic spectrum extends from λ = 0.7 to 1000 µm and is subdivided into different regions [5,8]. For example, it can be divided into the near infrared (NIR: 0.7–1.4 μm), SWIR: 1.4–2.5 μm and far infrared (FIR: 15–1000 μm) regions. The mid-wave infrared (MWIR) and long-wave infrared (LWIR) are the subsets that correspond to the wavelength ranges of 2.5−7 µm and 7−15 µm, respectively [9,10]. Spectral signals in MWIR and LWIR regions are produced as a consequence of molecular vibrations of the functional groups that can be related to mineralogy [11,12]. 

Numerous previous studies indicate that IR technologies can be utilised for the accurate identification of minerals. Such applications are usually qualitative. For example, near-infrared (NIR) sensors can provide accurate identification of clay minerals and rock-forming minerals [13,14], whereas short-wave infrared (SWIR) is one of the most widely used infrared technologies for the identification of alteration minerals [15,16]. On the other hand, LWIR permits identification of rock-forming minerals, whereas far-infrared (FIR) can be used for the identification of rare earth minerals [11,17]. Characteristic features of the minerals have also been utilised to quantitatively relate variations in mineral concentrations. For example, Hecker et al. [18] estimated concentrations of rock-forming minerals using LWIR. Similarly, Mroczkowska-Szerszeń and Orzechowski [19] used ATR-FTIR (attenuated total reflectance Fourier transform infrared) for semi-quantitative analysis of minerals in carbonate rocks. Palayangoda and Nguyen [20], on the other hand, estimated mineral concentrations in oil shale using ATR-FTIR spectra combined with Principal Component Regression (PCR) method. In another study, Guatame-Garcia and Buxton [21] assessed the use of infrared spectroscopy for predicting the soluble Al_2_O_3_ content in calcined kaolin. Although few researchers indicated the potential for using IR technologies in quantitative analysis of minerals, some authors also discussed the limitations of this approach. Specifically, Kaufhold et al. [22] assessed the possibility of the use of infrared spectra for quantitative analysis of clay minerals and pointed out the mineral-specific challenges owing to instrument detection limit, availability of suitable reference and particle size. At present, IR techniques are insufficiently used in quantitative analysis of minerals. Moreover, most of the existing studies in this field addressed the challenge for the development of reliable calibration models to predict mineral concentrations in complex mixtures. Consequently, there is a need for advanced data-driven approaches and spectral signal pre-processing techniques that can be incorporated into comprehensive calibration models, thus to achieve accurate estimation of mineral concentrations in different commodities. 

Data fusion is the term applied to the integrated analysis of multiple data blocks from different data sources, such that they can interact and inform each other [23]. Fusing of different data sources enhances the reliability of prediction or classification models owing to the synergy among the incorporated datasets. Data fusion can be implemented in different ways and at different levels using various multivariate linear (e.g., partial-least squares regression—PLSR) and non-linear (e.g., support vector machine—SVM) data analysis techniques [23,24]. Data fusion can be realised at low-, mid-, and high-level. In low-level fusion, data from the different sources are pre-processed and concatenated to form a fused data block [23,25]. Thus, it is commonly referred to as data-level fusion. The mid-level fusion requires two modelling steps. First, the informative features (relevant information) of the different data blocks are separately extracted using suitable variable screening or selection methods [23,26]. In the second step, the extracted features are concatenated into a single matrix and are used to develop models based on multivariate analysis techniques. In mid-level fusion, feature extraction can be accomplished using different strategies, such as data decomposition (Multivariate curve resolution—MCR) and feature selection (Principal Component Analysis—PCA) methods. Mid-level fusion is therefore also called features-level fusion. High-level fusion is a decision-level fusion, as the outputs (predicted value) of the prediction or classification models developed for each data block are fused (e.g., by averaging). Data fusion approaches are now widely used in several disciplines, such as robotics [27], image processing [28], food analysis [24,29,30], and pharmacological studies [26]. Findings yielded by pertinent studies indicate that data fusion approaches can be highly beneficial for mineralogical applications [31,32]. However, at present, the application of data fusion for mineralogical investigations remains very limited. 

The MWIR and LWIR provide spectral signals that can be used to identify various minerals. The LWIR permits identification of the rock-forming minerals. Whereas, the MWIR is the least-explored region of the electromagnetic spectrum, however, it has a great potential for material characterization. Therefore, combinations of the two regions can potentially result in a comprehensive and enhanced characterization of minerals. To date, quantitative analysis of minerals in polymetallic sulphide ore samples using MWIR and LWIR spectra combined with data fusion methods has never been conducted. This gap in the current analytical methodology and the promising findings [33] reported recently have motived the present study. Its main aims are thus (1) to investigate the use of diffuse reflectance infrared (MWIR and LWIR) spectra for quantitative analysis of mineral mixtures in polymetallic sulphide ore samples, and (2) to evaluate the data fusion methods using linear (PLSR and PCR) and non-linear (SVR) multivariate regression techniques. The implemented low-level data fusion approaches are data fusion without feature selection (fusion of the entire variables in the MWIR and LWIR data blocks) and with feature selection (fusion of the extracted features of the two data blocks). 

## 2. Materials and Datasets

### 2.1. Samples 

**Figure 1:**
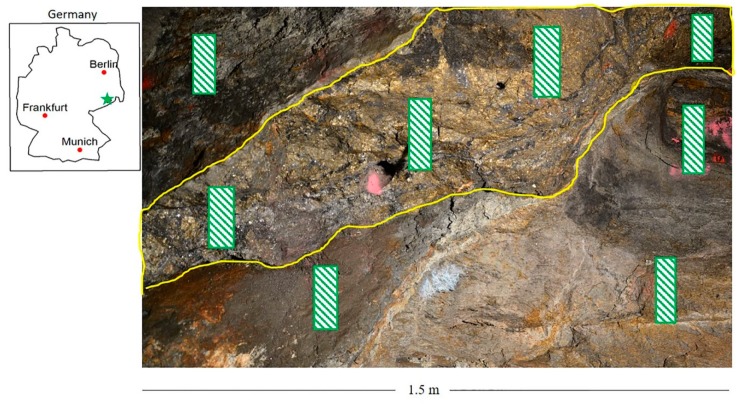
Location of the study site (the green star on the inset map) and underground mine face photo. The yellow line shows the boundaries of the ore zone. Outside of the ore zone, the host rock is gneiss. Some of the locations of the 58 samples used in this study are indicated in green boxes.

The study described in this paper is based on 58 representative rock samples collected from a polymetallic sulphide ore deposit formed by hydrothermal mineralisation processes. The typical ore minerals constituting the deposit are galena (PbS), pyrite (FeS_2_), sphalerite ((Zn, Fe)S), arsenopyrite (FeAsS), and chalcopyrite (CuFeS_2_), whereas associated gangue minerals include quartz (SiO_2_), barite (BaSO_4_), fluorite ((Ca, Ce, Y)F_2_), and carbonates (CO_3_^−2^) [34,35]. The samples were obtained from the ore and waste materials, which are sourced from different locations (Figure 1). A detailed description of the deposit type can be found in the work published by Desta and Buxton in 2018 [36]. 

### 2.2. Instrumentation and Datasets 

#### 2.2.1. Mid-Wave Infrared (MWIR) and Long-Wave Infrared (LWIR) Datasets 

The collected samples were powdered, and measurements were performed using the Agilent portable 4300 Fourier-transform infrared spectroscopy (FTIR) sensor. The FTIR infrared reflectance spectra required for the present investigation were acquired over the ~2.5 to ~15.0 µm wavelength range, as a mean of 64 sample scans at a resolution of 4 cm^−1^ using a diffuse reflectance-sampling interface. Samples heterogeneity was accommodated by collecting multiple spectra from each sample. Depending on the observed variability within each sample, 7 to 10 measurements were collected, and the averages were subsequently computed for each sample.

The acquired full-range FTIR dataset (covering the full wavelength span from 2.5 to 15.0 µm) were split into MWIR (2.5 to 7.0 µm) and LWIR (7.0 to 15.0 µm) spectral datasets. The full-range FTIR data were also analysed to compare the obtained results with the individual datasets and the data fusion outcomes. Therefore, the SiO_2_, Al_2_O_3_, and Fe_2_O_3_ composition prediction accuracy obtained using the three datasets (namely full-range FTIR, MWIR, and LWIR) and the fused datasets pertaining to 58 samples are discussed and compared in the sections that follow. 

#### 2.2.2. Chemical Analysis (XRF)

X-ray fluorescence (XRF) is a well-established technique for the analysis of chemical composition. It is an excellent method for determining the major and minor elements constituting whole rock. In this work, a conventional laboratory-based Malvern PANalytical Axios mAX wavelength dispersive X-ray fluorescence (WD-XRF) was used to acquire mineralogical information on SiO_2_, Al_2_O_3_, and Fe_2_O_3_ minerals. The detection limit of the XRF system is 0.01%. The quantitative mineralogical data obtained were employed in the validation of the developed methodological approaches.

## 3. Methodology 

### 3.1. Multivariate Analysis 

As a part of the exploratory data analysis, PCA was performed. Quantitative prediction of mineral concentrations in the polymetallic sulphide ore samples was achieved using both linear (PLSR and PCR) and non-linear (SVR) techniques. A brief description of these multivariate techniques is given below.

#### 3.1.1. Principal Component Analysis (PCA)

Principal component analysis (PCA) was performed to reduce data dimension by generating new sets of variables called Principal Components (PCs). This allows visualisation of multivariate data in a few PCs that are mutually orthogonal and thereby describe complementary information. In the present study, PCA was applied to the individual datasets as well as to the fused spectral data. The scores and loading plots of the PCA models were used to investigate sample−variables relationships and the grouping structure (intra-sample relationships). 

#### 3.1.2. Partial-Least Squares Regression (PLSR)

Partial least-squares regression (PLSR) is a multivariate data analysis technique that maximises the covariance between the predictor (X) and the response (Y) matrices. It models the response and predictor matrices simultaneously to find the latent variables in the predictor (X) that will best predict the latent variables in the response (Y). PLSR generates principal components (PCs) that explain the variation in X that correlates to the variation in Y [37]. While PCA is utilized to extract PCs that describe variations in the data, PLSR allows PCs to be correlated with the response (Y) to compute latent variables (LVs). Thus, the LVs in X can be used to predict the LVs in Y. LVs are important factor in determining model performance. In this work, the dependent variables (the response) are mineral concentrations and the independent variables (the predictors) are the IR spectra (e.g., MWIR and fused data blocks). The PLSR models were developed using both individual and fused data blocks. The calibration datasets were used to develop the PLSR models and their predictive performance was validated using independent datasets (validation datasets). 

#### 3.1.3. Principal Component Regression (PCR)

Principal component regression (PCR) is a regression technique that relates the variance in a response variable (Y) to the variance in several predictors (X variables). As PCR is a two-step method, the X-matrix (comprising of X variables) is decomposed using PCA [38,39,40]. In the second step, the PC scores (instead of the original X variables) are used as predictors to fit a multiple linear regression (MLR) model, aiming to establish a linear relationship between the predictor (X variables) and the response (Y variable) using the typical least squares procedure [41]. As the PCR is based on the orthogonal scores, the model does not suffer from collinearity effects. Unlike in the PLSR, the response variable in PCR plays no role in identifying the PCs’ directions. In the present study, the PCR models were developed using the IR spectra (individual MWIR and LWIR, as well as fused data blocks) as the predictor and the mineral concentrations as the response variables. The Singular Value Decomposition (SVD) algorithm was used to calculate the PCs of the PCR models. The weights of X variables and the Y variables were standardised. 

#### 3.1.4. Support Vector Regression (SVR)

Support vector machine (SVM) is a supervised learning algorithm for the analysis of classification (support vector classification—SVC) and regression (support vector regression—SVR) problems [42,43]. SVM maps the input data into a higher-dimensional feature space using kernel functions, which can take many forms, such as linear, polynomial, radial basis function (RBF), sigmoid, etc. Therefore, SVM is a powerful technique that can be applied to both linear and non-linear systems. Detailed theoretical background on SVR can be found in pertinent literature [42,43,44,45]. In this study, three different kernel functions (RBF, sigmoidal, and polynomial) were examined and the optimal kernel function was selected based on the RMSE and R-squared values. As result, RBF kernel function was selected. RBF can be utilised to model non-linear systems of varying complexity. The SVM regression type used in this work is ε-SVR with RBF kernel function. The key model parameters for the specification of ε-SVR models are C value and epsilon (ε), as they respectively determine the trade-off between the training error and the model complexity (flatness), and control the width of the band where the cost of errors in the epsilon-intensive loss function is zero. The value of ε can thus affect the number of support vectors (SVs) used to construct the regression function. The ε-SVR models developed as a part of this work use the IR spectra (comprising the individual and fused datasets) as the input vector and mineral (SiO_2_, Al_2_O_3_, and Fe_2_O_3_) concentrations as the response vector. As in SVM the values of the optimal model parameters are not known in advance, C and ε were optimised using grid search approach with a leave-one-out cross-validation. 

### 3.2. Model Performance Assessment

The performance of the prediction models was investigated using root mean square error of cross validation (RMSECV), root mean square error of prediction (RMSEP), and the correlation coefficient (R^2^). In RMSECV, the error on test split is calculated using a cross-validation scheme; however, performance is based on the calibration cases. In this work, the RMSECV corresponds to the results of a leave-one-out cross-validation that prevents model over-fitting. Specifically, when calculating the RMSECV value, each sample was removed at a time from the calibration data and the models were built using the remaining data in the training set. Performance of each of these resulting models was validated using the removed sample. The process was repeated until each sample in the training dataset has been removed once. The RMSECV was used to select the optimal number of PCs in the PLSR and PCR models, and to specify model parameters in SVR. RMSEP represents the prediction error based on a comparison of real cases not used to make the model with reference values (in this case, an independent dataset). Consequently, RMSEP indicates how well the model built using calibration data performs when applied to unknown cases. R^2^ denotes the strength of the linear relationship between the response and predictor variables. When R^2^ is computed using the validation samples it signifies a model’s predictive ability. Improved predictive performance is associated with a lower value of statistical error terms (RMSECV and RMSEP) and a higher predicted R^2^.

### 3.3. Data Pre-Processing 

Infrared measurements include undesired variations (e.g., instrumental artefacts), which are generally compensated by data pre-processing, whereby unwanted variation within the data is removed to enhance the signal pertaining to the analytical information [46,47]. The choice of data filtering techniques adopted for this purpose affects the outcome. Therefore, design of experiment (DoE) is required to identify the optimal data pre-processing techniques that yield the best results (in this case, mineral concentrations prediction). In the present study, DoE was developed considering mean centring (MC) and the signal correction methods, namely baseline correction, normalisation, standard normal variate (SNV) and smoothing (Gaussian filter smoothing) data pre-processing techniques. These methods were chosen, as the aim was to remove the most common artefacts from the infrared spectra (e.g., baseline shift).

MC is a data scaling technique that can be adopted to remove offsets by subtracting the variable mean from each value [46,48]. Baseline correction subtracts the unwanted “background signal” from each spectrum [46]. The aim of normalisation is to divide each spectrum based on the estimation of its spectral intensity and remove undesired intensity variation due to multiplicative effects [46]. SNV minimises the light scattering effect and particle size effects in the spectra data. It is employed to normalise the spectrum by subtracting its mean value from each variable and dividing the resulting variables by the spectrum standard deviation [46,49]. Finally, smoothing allows random noise to be removed from the dataset by averaging the neighbouring points [46]. The signal correction methods are performed on one sample at the time (row-wise), whereas for mean centring, the pre-processing is applied to individual columns. 

### 3.4. Data Fusion 

**Figure 2:**
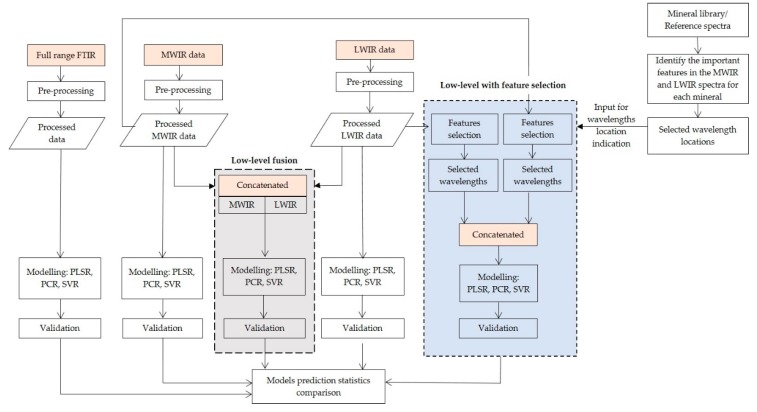
Workflow diagram depicting the steps of the low-level fusion (1) without feature selection (the grey box) and (2) with feature selection (the blue box).

Integration of data blocks from multiple sensors can enhance prediction accuracy and support better interpretation of model outputs. Data fusion requires pre-processing of the individual datasets, ultimate multivariate data analysis method and robust correlation of the dependent and independent variables [23]. The schematic diagram of the data fusion method adopted in this work is provided in Figure 2. As can be seen, the pre-processed datasets and the three multivariate techniques (PLSR, PCR, and SVR) were used for the realisation of low-level fusion of the MWIR and LWIR data blocks without feature selection and with feature selection (the grey and blue boxes of Figure 2, respectively). The methodological approaches applied for the implementation of the two data fusion approaches are described below. 

#### 3.4.1. Low-Level Data Fusion without Feature Selection 

Depending on the dataset or detector, the amount and type of noise might differ across the IR range. Therefore, pre-processing of the individual data blocks, separately, allows investigating and treating the various noise sources across the two IR (MWIR and LWIR) wavelength ranges, independently. In the low-level fusion without feature selection approach, the individual pre-processed reflectance spectra acquired from the MWIR and LWIR data sources were concatenated into a single matrix, as shown in the grey box of Figure 2. Therefore, four fused data blocks were generated, corresponding to the application of four pre-processing techniques (SNV, normalise, baseline, and smoothing) to the individual data blocks (MWIR and LWIR). The fused data blocks were used to develop the prediction models using PLSR, PCR, and SVR algorithms, as shown in Figure 2. The models were developed using the training (calibration) datasets and were subsequently validated using the independent (validation) datasets. 

#### 3.4.2. Low-Level Data Fusion with Feature Selection

Unlike low-level fusion, mid-level data fusion requires features reduction, which is achieved through variable screening, and thus allows all non-informative variables to be removed in the feature selection step. The mid-level fusion requires a modelling step for the extraction of the informative features. However, the feature selection method deployed in this study was not based on models’ outputs. Thus, the approach is not a standard mid-level fusion where modelling is involved for the extraction of the important variables. Therefore, it is referred to as a low-level fusion with feature selection. 

In the low-level fusion with feature selection approach, informative features (in this case, those that contain information pertinent for the prediction of mineral compositions of interest) were independently extracted from the MWIR and LWIR data blocks. The variable selection or feature extraction technique used in this study is based the reference spectra of the minerals from the well-established mineral spectral libraries. Feature selection requires highly efficient data reduction methods, as the aim is to retain only the most important variables in the model. The mineral libraries show the infrared reflectance spectra of the (relatively pure) minerals and were used to identify the wavelength locations of the spectral features corresponding to the functional groups of the target minerals (e.g., Si−O). In this work, the hypothesis for the low-level fusion with feature selection implementation is that variables that correspond to the main spectral features are the most informative for the prediction of mineral concentrations. Therefore, variable screening was performed based on a prior knowledge-based approach. 

The pre-processing techniques described in Section 3.3 were applied on the individual datasets prior to variable screening. Subsequently, the important variables (relevant information related to the chemical composition) were retrieved from both MWIR and LWIR pre-processed data blocks separately. The extracted features from the two data blocks were aligned and concatenated into a single matrix. Therefore, the most relevant variables that explain most of the variations in the spectra were fused and mean centred. Prediction models were developed using the fused data blocks comprising of the extracted features and the three multivariate regression techniques (PLSR, PCR, and SVR). The workflow of the low-level fusion with feature selection approach is presented in the blue box of Figure 2. 

#### 3.4.3. Individual Datasets 

The prediction models were developed using the individual data blocks (MWIR and LWIR) and the three aforementioned analytical techniques (PLSR, PCR, and SVR). The Y (response) variables are the concentrations of the minerals (SiO_2_, Al_2_O_3_, and Fe_2_O_3_). A series of models were developed using the pre-processed MWIR and LWIR data separately. The prediction performance of each model was evaluated using independent validation datasets. Next, performance of the prediction models based on the fused datasets was compared with that of the models developed using individual data blocks (MWIR and LWIR). In the present study, the MWIR and LWIR spectral data were acquired using a single instrument (physically integrated system). Thus, to assess the performance of the full-range FTIR data model with the fused and individual data blocks, prediction models were developed using the full-range FTIR data. The main difference between the full-range FTIR data and the low-level fusion is the later pre-processed the individual datasets separately and concatenated. Whereas, the former considers both ranges (the MWIR and LWIR) in the pre-processing stage. The low-level fusion approach is useful in treating different forms of noise in the spectra data block by data block. Finally, the prediction performances of the models developed using the individual techniques, the full-range FTIR and the two low-level data fusion approaches were assessed based on the RMSECV, RMSEP, and R^2^ values.

### 3.5. Calibration and Validation Datasets 

The 58 samples that were analysed were divided into calibration and validation subsets using a random sample selection algorithm, which was first applied to the MWIR dataset. The randomly selected samples were assigned into the calibration and validation datasets of the full-range FTIR and LWIR datasets. The same procedure was followed for the three datasets (Si_2_O_3_, Al_2_O_3_, and Fe_2_O_3_) to ensure that all models related to each mineral utilise the calibration and validation datasets comprising of the same samples. The calibration dataset consisted of 43 sample measurements and the validation dataset included 15 remaining measurements. To allow a direct model comparison, the same split was maintained in the calibration and validation datasets of the individual data blocks (MWIR and LWIR), the full-range FTIR dataset, and the fused datasets. In this study, all the analyses were performed using the Unscrambler and R software. 

## 4. Results and Discussion 

### 4.1. The Individual Datasets 

#### 4.1.1. Spectra Features of the Minerals 

**Figure 3:**
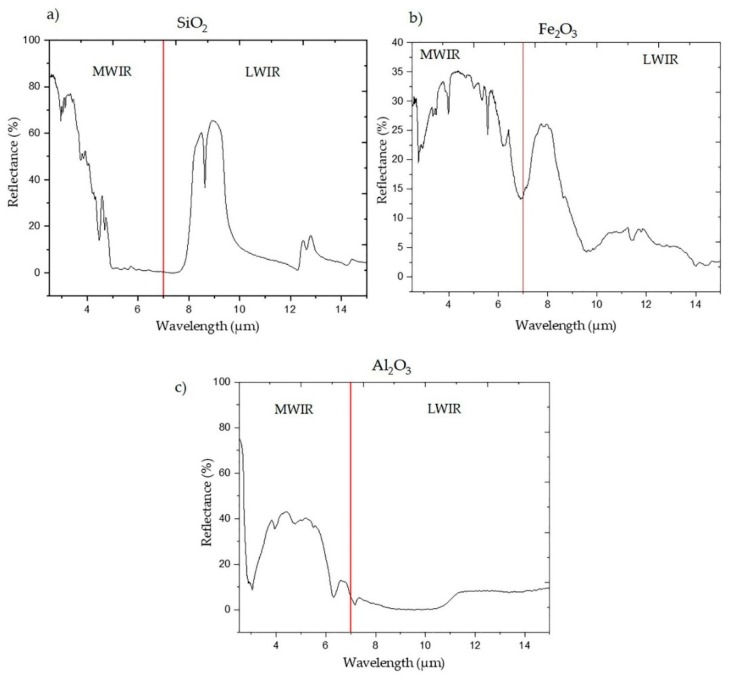
The mid-wave infrared (MWIR) and long-wave infrared (LWIR) reflectance spectra of (**a**) SiO_2_; (**b**) Fe_2_O_3_; and (**c**) Al_2_O_3_ (Source: Ecostress Spectral library [50]).

Typical MWIR and LWIR spectra of nearly pure SiO_2_, Al_2_O_3_, and Fe_2_O_3_ are shown in Figure 3. In the MWIR region, the Al_2_O_3_ spectrum exhibits significant features at 2.9 µm, 3.97 µm, 4.75 µm, and 6.28 µm wavelengths. In the LWIR region of the SiO_2_ spectrum, stretching vibration modes can be seen in the 8−10 µm and 12–14 µm regions due to Si-O stretching. Fe_2_O_3_ spectrum similarly shows prominent spectral features (peaks) at 3.45 µm, 3.97 µm, 5.57 µm, and 6.76 µm. The spectra pertaining to the three minerals show important features (prominent peaks) that are caused by the molecular vibration of the functional groups of each mineral. Therefore, it is likely that the mineral concentrations can be related to the reflectance value of each sample’s spectrum. 

#### 4.1.2. Exploratory Analysis 

**Figure 4:**
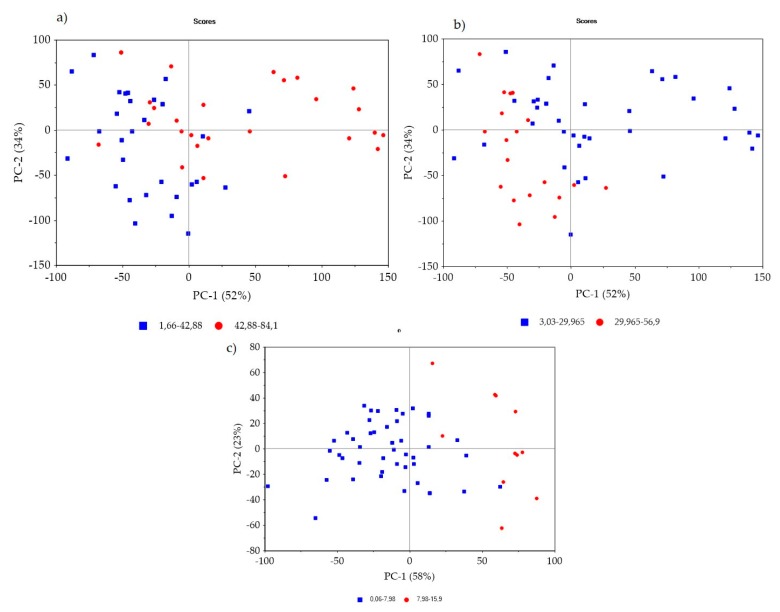
Principal component analysis (PCA) model score plots of (**a**) SiO_2_; (**b**) Fe_2_O_3_; and (**c**) Al_2_O_3_ concentrations categorized into two ranges (the concentrations are expressed in wt %).

Mineral concentrations varied greatly among the analysed samples, as the Fe_2_O_3_ value ranged from 3.03 to 59.9 wt% with a mean of 24.61, whereas the SiO_2_ value ranged from 1.66 to 84.1 wt% with a mean of 41.28 wt%, and 0.06−15.9 wt% (M = 4.22 wt%) was obtained for Al_2_O_3_. Figure 4 shows the PCA model score plots of the full-range FTIR data for the SiO_2_, Fe_2_O_3_, and Al_2_O_3_ datasets. The plots provide information on the potential patterns that are related to the mineral’s concentration. 

#### 4.1.3. MWIR and LWIR Data Models

In Table 1, Table 2 and Table 3, the calibration and prediction statistics of the five datasets for Fe_2_O_3,_ SiO_2_, and Al_2_O_3_ prediction, respectively, are summarised. The prediction models were developed once each dataset has been subjected to the data pre-processing techniques mentioned in Section 3.3. However, the prediction performance of the data models declined after SNV filtering and not showed significant improvement after data smoothing. Therefore, the tabulated data indicate model performance after normalisation and baseline correction have been applied to the datasets. 

It is evident that a more accurate prediction was obtained by applying the data pre-processing techniques to MWIR, LWIR, and full-range FTIR datasets. For example, for Al_2_O_3_ prediction using PLSR, the normalized MWIR data model resulted in an improved performance than the raw MWIR data model (Table 3). Similarly, the prediction performance of both PCR and SVR models improved after data pre-processing (Table 1, Table 2 and Table 3). For all three models (PLSR, PCR, and SVR) used to predict Fe_2_O_3_ and SiO_2_, the error terms declined and the R^2^ value improved after MWIR data normalisation. Likewise, the LWIR data models developed using the three algorithms (PLSR, PCR, and SVR) exhibited improvement after data pre-processing. For example, for the prediction of SiO_2_ concentration, the normalized LWIR data model showed a remarkable improvement than the raw LWIR data model (Table 2). 

As can be seen from the results reported in Table 1, Table 2 and Table 3, normalisation of the IR data resulted in remarkable improvement in the performance of all models, suggesting presence of undesired intensity variations in the spectra caused by multiplicative effects. On the other hand, not all data filtering techniques necessarily improved model performance, as was the case for the SNV filtering technique, irrespective of the dataset or multivariate regression method (PLSR, PCR, or SVR) used. This is most likely due to the minimal effects of light scattering and particle size in the IR spectra of the analysed samples. Combination of the pre-processing techniques were analysed for the prediction of the mineral’s concentration, however, the prediction performances of the models were not improved, thus the results are not included in this paper. 

It is also evident that the prediction performance of models based on MWIR and LWIR data depends on the mineral type. For example, LWIR-based models outperform those utilising MWIR data in the quantification of the SiO_2_ concentration (Table 2). Conversely, MWIR data models yielded more accurate Al_2_O_3_ concentration prediction (RMSEP = 1.86, R^2^ = 0.85) than those based on LWIR (RMSEP = 2.14, R^2^ = 0.8), as shown in Table 3. It is likely that prediction accuracy is linked to the amount of spectral information (relevant spectral features) in the IR dataset. For example, as shown in Figure 3 and discussed in Section 4.1.1, the Al_2_O_3_ spectrum contains more informative spectral features in the MWIR region than in the LWIR region. Conversely, the SiO_2_ spectrum shows a greater number of prominent spectral features in LWIR than in the MWIR region (Figure 3), thus resulting in superior prediction of SiO_2_ concentration by the model based on LWIR data. 

As shown in Figure 3, the pure minerals show spectral features in both MWIR and LWIR regions. However, the spectrum of each sample also includes information pertaining to the complex matrix of sulphide minerals, making identification of each individual component challenging. For this reason, in this work, three multivariate analysis techniques (PLSR, PCR, or SVR) were adopted, confirming that semi-quantification of the minerals in a polymetallic sulphide ore samples was possible using individual MWIR and LWIR datasets. This is an interesting finding, since the MWIR region of the electromagnetic spectrum is rarely used in lithological material characterisation.

### 4.2. Low-Level Fusion without Feature Selection 

**Figure 5:**
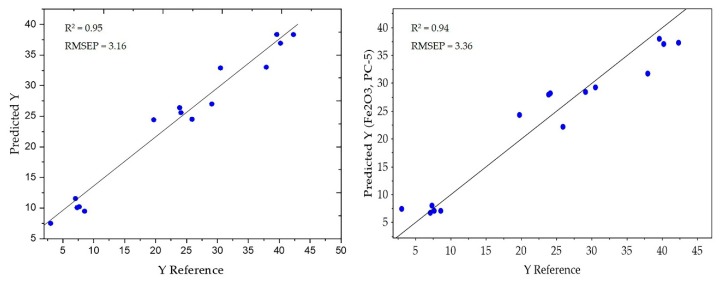
(**a**) SVR; and (**b**) PCR regression results for the predicted vs. actual Fe_2_O_3_ concentration after applying low-level fusion on the normalised MWIR and LWIR data blocks.

In low-level data fusion, data integration occurs in the initial stages of the analytical data flow, after proper pre-processing [23]. Thus, mineral concentration prediction based on this approach is highly influenced by the choice of pre-processing techniques. In the present study, as shown in Table 1, a better prediction of Fe_2_O_3_ concentration (RMSEP = 3.31, R^2^ = 0.94) was achieved using the PLSR model when the normalised MWIR and LWIR data blocks were fused than when these datasets were treated with SNV (RMSEP = 4.76, R^2^ = 0.87). Moreover, the SVR model resulted in a better prediction of Fe_2_O_3_ after normalisation (RMSEcv = 3.90, RMSEP = 3.16, R^2^ = 0.95) relative to that yielded by the PLSR or PCR models (Table 1 and Figure 5). 

**Figure 6:**
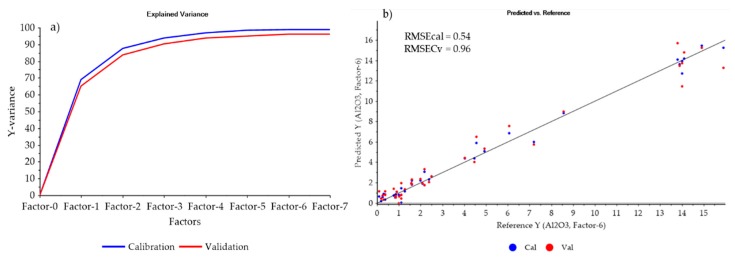
PLS regression results based on the dataset formed by low-level fusion of the normalised MWIR and LWIR data blocks for predicting Al_2_O_3_ concentrations (**a**) the explained variance (**b**) the predicted vs. actual concentration for the calibration (RMSEcal) and cross-validation (RMSEcv) models.

Similarly, enhanced SiO_2_ prediction was achieved after the normalised MWIR and LWIR data blocks were fused compared to the outputs produced using other pre-processing techniques (Table 2). For the prediction of Al_2_O_3_, low-level fusion of normalised MWIR and LWIR data blocks resulted in a better prediction than when the data blocks were treated with the other data filtering techniques (Table 3, Figure 6, and Table S3). These findings confirm the need for adopting DoE in the selection of most optimal data filtering techniques. 

As noted in Section 2.2.1, the MWIR and LWIR datasets were acquired using a single-sensor FTIR spectrometer. This allowed the performance of models based on the full-range FTIR data (which includes both MWIR and LWIR datasets) to be assessed and compared to the low-level fusion results. The findings revealed that the prediction models applied to the dataset formed by low-level fusion are superior to the full-range FTIR data models. For example, for the prediction of Fe_2_O_3_, the optimal PLSR model after low-level fusion has an RMSEP = 3.3 and R^2^ value of 0.94, compared to RMSEP = 3.68 and R^2^ = 0.92 obtained for the full-range FTIR (Table 1). Similarly, using low-level fusion for the prediction of SiO_2_ and Al_2_O_3_ concentration is superior to the results obtained using the full-range FTIR data (Table 2 and Table 3). This might be due to the different amount of noise in the MWIR and LWIR wavelength regions that require independent pre-processing of the two data blocks. Even though these improvements are not statistically significant, the results suggest data fusion as a better and comparative option for a combination of multiple sensors. This is an interesting point, since the physical integration of multiple sensors into a single platform is challenging and expensive, in terms of practical implementation. Thus, for a combination of multiple data sources, data fusion can be considered as an economic and practical alternative option.

### 4.3. Low-Level Data Fusion with Feature Selection

In this study, the extracted informative variables from the two data blocks are indicated in Table 4. The prediction of Al_2_O_3_ concentration using PLSR and the low-level fusion with the selected features after data normalization, significantly improved compared to applying the models to datasets subjected to low-level fusion without feature selection as well as the full-range FTIR data models (Table 3). Similarly, after low-level fusion with the selected features, enhanced Al_2_O_3_ prediction performance was observed for models based on the PCR and SVR (Table 3). These findings indicate that the feature selection approach was able to capture most of the important variations in the spectral data. In addition, by excluding the irrelevant information, feature selection method enhanced the prediction performance of the Al_2_O_3_ models.

**Figure 7:**
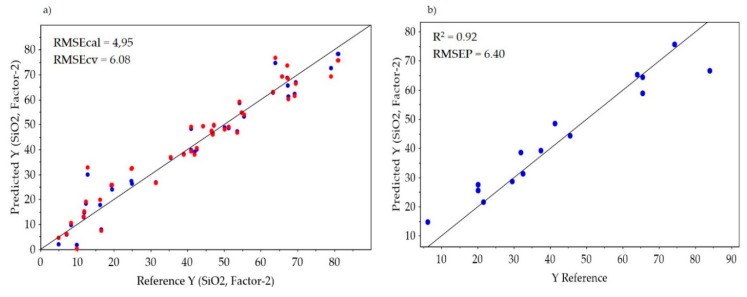
PLS regression of predicted vs. actual SiO_2_ concentration after the selected features fusion of the normalised MWIR and LWIR data blocks (**a**) for calibration and cross-validation; and in (**b**) the prediction model.

The SiO_2_ and Fe_2_O_3_ prediction models after the selected features fusion were better than the individual datasets models (Table 1, Table 2, and Figure 7). However, low-level fusion without feature extraction resulted in a better Fe_2_O_3_ and SiO_2_ concentration prediction relative to the extracted features fusion (Table 1 and Table 2). This is likely due to the fact that not all relevant information was retained in the extracted spectra of the minerals. Therefore, alternative feature extraction techniques (e.g., multivariate curve resolution-MCR) can likely improve the fusion results. 

The main advantage of feature selection (variable screening) is that non-informative variation can be removed in the variable screening step, potentially enhancing the prediction accuracy. The rapid advances in sensor technologies allow generation of multi- and mega-variate data. These datasets can be utilised in data-driven approaches. Nonetheless, high data volume remains a significant challenge for both data processing and storage. Therefore, data volume reduction without loss of information is always preferable. This can be achieved using multivariate data analysis techniques and data fusion approaches. For example, in this work, when variable screening was performed prior to the implementation of the low-level data fusion, data volume reduction from 79% to 58% was achieved. Specifically, for the prediction of Fe_2_O_3_ and Al_2_O_3_ concentration 21% and 40% of the variables (data) were used, respectively, in the prediction models to retain the important information while enhancing prediction accuracy (Tables S1–S3).

### 4.4. Data Fusion vs. Individual Sensors

Despite the fact that IR technologies are mainly used for qualitative analysis of materials, the results obtained in this work show the potential of the individual techniques (MWIR and LWIR) for quantitative analysis of minerals in polymetallic sulphide ore samples. Moreover, data fusion both with and without feature selection yielded better prediction performance compared to those based on individual techniques and the full-range FTIR data models (Table 1, Table 2 and Table 3). This is likely due to the fact that the fused data blocks use the synergy between the two data blocks (MWIR and LWIR). In addition, extraction of the informative variables maximizes the relevant information (related to the concentration of the minerals) in the fused data models. Therefore, data fusion is a preferred approach for quantitative analysis of minerals. 

It is also worth noting that some of the models based on individual (MWIR and LWIR) datasets yielded more accurate prediction than did models based on the full-range FTIR dataset. For example, applying models based on PLSR, PCR, and SVR on the LWIR data resulted in enhanced SiO_2_ prediction compared to the full-range FTIR model (Table 2). This indicates the importance of extracting the informative variables from the two datasets prior to modelling, which was achieved in this work by adopting data fusion.

Data fusion allows handling different forms of uncertainties (e.g., different forms of noise) prior to modelling and is thus very useful approach for both classification and prediction problems analysis using various classification or regression algorithms. Its main benefits are enhanced prediction accuracy, lower uncertainty, enhanced availability of information, and holistic description of materials under investigation. Moreover, the physical integration of sensors requires complex and expensive system design. Therefore, data fusion is a promising alternative for enhanced characterisation of materials in mining operations using multiple sensors. 

### 4.5. Comparison of the Proposed Models 

In the present study, adoption of linear and non-linear multivariate techniques (PLSR, PCR, and SVR) resulted in comparable performance in terms of prediction of the minerals concentrations. Particularly, the PLSR and PCR results are similar. The major difference was obtaining the higher number of factors (PCs) for PCR (Tables S1–S3). In general, the overall results show both the linear and non-linear techniques provided good and acceptable results. Therefore, for the given datasets, moderate effects of the choices of models (linear or non-linear models) were observed. 

### 4.6. Benefits and Limitations of the Proposed Approach for Mining Applications 

The results reported in this work demonstrate that MWIR and LWIR spectral ranges capture information relevant for predicting mineral concentrations in polymetallic sulphide ore samples. While data fusion appears to enhance model prediction accuracy, it may be difficult to apply to data obtained from multiple sources. A further potential challenge stems from the large data matrix produced by data concatenation, as this is likely to cause both computational and data storage issues. However, fusion of the extracted informative variables minimises the data volume using variable screening and was shown in this work to yield enhanced or comparable prediction performance. This is an interesting finding, since it shows the potential of the proposed approach for integration of multiple data sources (such as SWIR or Raman spectra) without generating a large data matrix after concatenation. 

Quantified mineralogical information is crucial for elucidating the variability within a deposit, and can benefit in geometallurgical characterisation (e.g., different minerals have different flotation properties), controlling ore grade, defining blasting parameter requirements, and ensuring product quality. Thus, it can be highly valuable for maximising the potential economic benefit of mining operations. Currently, quantitative analysis of minerals is conducted using X-ray diffraction (XRD) or automated scanning electron microscopy (ASEM), both of which are laboratory-based techniques. Thus, IR systems coupled with data fusion approaches can be considered as complementary techniques to achieve rapid determination of mineral concentrations. Overall, the prediction accuracies achieved in this study are sufficient for rapid in-situ indication of mineral concentrations in polymetallic sulphide ores using a portable system. Therefore, the availability of the portable instruments combined with the promising results of this study supports the practicality of the proposed approach for online in-situ analysis of minerals. 

## 5. Conclusions 

In this work, different scenarios were investigated to assess their influence on the prediction of SiO_2_, Al_2_O_3_ and Fe_2_O_3_ concentrations in polymetallic sulphide ore samples using infrared reflectance spectra, namely:

(1) the use of individual spectral regions (MWIR and LWIR);
(2) the effect of different data pre-processing techniques on the prediction performance;
(3) potential for improvement in prediction accuracy by applying low-level and low-level with feature selection data fusion approaches;
(4) comparative benefits of applying linear (PLSR and PCR) and non-linear (SVR) multivariate analysis techniques.

The results reported in the preceding sections show that both MWIR and LWIR datasets include relevant information that can be employed in determining mineral concentrations. Moreover, data fusion significantly improved model prediction accuracy. Models incorporating both the linear and non-linear multivariate techniques (PLSR, PCR, and SVR) resulted in comparable performance. The choice of the data pre-processing techniques was shown to exert significant influence on the model output. For the prediction of Al_2_O_3_, the best-performing model was achieved using PLSR and the low-level fusion of the extracted features after data normalisation (RMSEP = 1.4, R^2^ = 0.91). The PLSR model better predicted Fe_2_O_3_ in polymetallic sulphide ore after low-level fusion of normalised MWIR and LWIR data blocks (RMSEP = 3.3, R^2^ = 0.94). Finally, the best prediction of SiO_2_ concertation was achieved by the PLSR model after normalised data blocks were subjected to low-level fusion (RMSEP = 5.96, R^2^ = 0.93). Overall, both the linear and non-linear techniques provided good and acceptable results. Although the acquired prediction accuracies are lower than those of the standard laboratory-based techniques, the proposed method is suitable for rapid in-situ indication (semi-quantification) of mineralogical concentrations along the mining value chain.

The fact that the use of the extracted features significantly reduced the data volume and resulted in promising results suggests a great potential of applying data fusion to data obtained from multiple sources. Our future work will focus on extending the data fusion framework for integration of additional data sources (e.g., SWIR and Raman) to achieve a holistic description and improved quantification of minerals in different deposit types using the synergy among the different data sources. This will be beneficial for improving resource efficiency in the mining industry. 

## Figures and Tables

**Table 1:** Statistical summary of the partial-least squares regression (PLSR), principal component regression (PCR), and SVR models for the prediction of Fe_2_O_3_. The concentrations of Fe_2_O_3_ in the analysed samples were in the range of 3.03−59.9 wt%.

Datasets/Fusion Method	Pre-Processing	PLSR		PCR		SVR	
		RMSEP	R^2^	RMSEP	R^2^	RMSEP	R^2^
MWIR	Raw	6.18	0.78	7.88	0.64	5.50	0.81
	Normalize	4.53	0.88	4.97	0.86	3.95	0.90
	Baseline	5.02	0.86	4.01	0.91	6.39	0.77
LWIR	Raw	7.32	0.69	5.97	0.80	4.78	0.85
	Normalize	4.51	0.88	5.34	0.84	4.57	0.87
	Baseline	7.50	0.68	5.79	0.81	5.26	0.84
Full-range	Raw	6.05	0,79	5.2	0,84	4.71	0.87
	Normalize	3.68	0,92	3.95	0.91	3.40	0.93
	Baseline	4.29	0.89	4.03	0.91	4.86	0.87
Low-level	Normalize	3.30	0.94	3.36	0.94	3.16	0.95
	Baseline	4.57	0.88	3.87	0.91	4.94	0.84
Low-level with the selected features	Normalize	4.22	0.90	4.44	0.89	4.34	0.89
	Baseline	5.18	0.85	5.76	0.81	7.34	0.69

**Table 2:** Statistical summary of the PLSR, PCR, and SVR models for the prediction of SiO_2_. The concentrations of SiO_2_ in the analysed samples were in the range of 1.66−84.1 wt%.

Datasets/Fusion Method	Pre-Processing	PLSR		PCR		SVR	
		RMSEP	R^2^	RMSEP	R^2^	RMSEP	R^2^
MWIR	Raw	7.95	0.87	8.22	0.86	10.30	0.74
	Normalize	7.77	0.88	8.80	0.84	8.47	0.86
	Baseline	8.40	0.86	7.38	0.89	9.89	0.82
LWIR	Raw	12.8	0.67	9.69	0.81	9.13	0.83
	Normalize	6.12	0.92	6.50	0.91	6.56	0.90
	Baseline	9.13	0.83	9.06	0.83	8.74	0.85
Full-range	Raw	6.95	0.90	7.55	0.88	9.14	0.86
	Normalize	6.42	0.92	7.16	0.90	7.52	0.90
	Baseline	7.19	0.90	8.44	0.86	9.08	0.83
Low-level	Normalize	5.96	0.93	7.17	0.90	6.85	0.90
	Baseline	7.66	0.88	8.56	0.85	8.69	0.89
Low-level with the selected features	Normalize	6.40	0.92	6.06	0.93	6.77	0.91
	Baseline	8.30	0.86	8.37	0.86	10.10	0.81

**Table 3:** Statistical summary of the PLSR, PCR, and SVR models for the prediction of Al_2_O_3_. The concentrations of Al_2_O_3_ in the analysed samples were in the range of 0.06−15.9 wt%.

Datasets/Fusion Method	Pre-Processing	PLSR		PCR		SVR	
		RMSEP	R^2^	RMSEP	R^2^	RMSEP	R^2^
MWIR	Raw	2.16	0.79	2.05	0.81	1.69	0.86
	Normalize	1.86	0.85	1.92	0.84	1.93	0.83
	Baseline	2.11	0.80	1.99	0.82	1.68	0.88
LWIR	Raw	2.47	0.73	2.59	0.70	2.3	0.77
	Normalize	2.09	0.80	2.03	0.82	1.86	0.85
	Baseline	2.29	0.76	2.71	0.75	1.83	0.84
Full-range	Raw	2.02	0.82	1.99	0.82	1.75	0.87
	Normalize	2.02	0.82	1.99	0.82	1.9	0.85
	Baseline	2.15	0.79	1.82	0.85	1.69	0.87
Low-level	Normalize	1.95	0.83	2.06	0.81	1.83	0.86
	Baseline	2.06	0.81	2.13	0.80	1.68	0.88
Low-level with the selected features	Normalize	1.40	0.91	1.48	0.90	1.79	0.86
	Baseline	1.82	0.85	1.77	0.86	1.59	0.89

**Table 4:** The wavelength range of the features related to SiO_2_, Al_2_O_3_, and Fe_2_O_3_ mineral composition extracted from the MWIR and LWIR reflectance datasets.

Minerals	MWIR Wavelength (µm)	LWIR Wavelength (µm)
Al_2_O_3_	2.85–3.10	7.00–7.29
	3.83–5.73, 6.20–6.40	10.50–11.40
Fe_2_O_3_	2.78–2.92, 3.38–3.5, 3.92–4.03, 5.0–5.10	7.00–7.20, 7.74–8.05, 9.38–10.00
	5.30–5.39, 5.53–5.69, 6.15–6.31, 6.76–7.00	11.30–11.6, 13.90–14.10, 14.40–14.60
SiO_2_	3.65–4.93	8.00–10.00
		12.00–13.00

## Supplementary Materials

The following are available online at https://www.mdpi.com/1424-8220/20/5/1472/s1, Table S1: Statistical summary of the PLSR, PCR and SVR models for the prediction of Fe_2_O_3_. The concentrations of Fe_2_O_3_ in the analysed samples were in the range of 3.03–59.9 wt%, Table S2: Statistical summary of the PLSR, PCR and SVR models for the prediction of SiO_2_. The concentrations of SiO_2_ in the analysed samples were in the range of 1.66–84.1 wt%, Table S3: Statistical summary of the PLSR, PCR and SVR models for the prediction of Al_2_O_3_. The concentrations of Al_2_O_3_ in the analysed samples were in the range of 0.06–15.9 wt%.
