# Supplementary material for: Fusion of Mid-Wave Infrared and Long-Wave Infrared Reflectance Spectra for Quantitative Analysis of Minerals
Source: Sensors (Basel). 2020 Mar 7;20(5):1472. doi: 10.3390/s20051472 (PMC7085633; doi:10.3390/s20051472)
Supplement: Supplementary file 1 [file sensors-20-01472-s001.pdf]

Table S1. Statistical summary of the PLSR, PCR and SVR models for the prediction of Fe_2O_3. The concentrations of Fe_2O_3 in the analysed samples were in the range of 3.03–59.9 wt%.

Datasets/ Fusion method	No. of Variables	Pre-Processing	PLSR				PCR				SVR		
			Calibration	Validation		Factors	Calibration	Validation		Factors	Calibration	Validation	
			RMSEcv	RMSEP	R^2		RMSEcv	RMSEP	R^2		RMSEcv	RMSEP	R^2
MWIR	5520	Raw	5.36	6.18	0.78	5	5.9	7.88	0.64	7	8.1	5,5	0,81
		Normalize	5.9	4.53	0.88	4	6.47	4.97	0.86	5	5.01	3.95	0,9
		Baseline	6.01	5.02	0.86	5	5.01	4.01	0.91	9	9.17	6.39	0,77
LWIR	1672	Raw	6.98	7.32	0.69	5	6.5	5.97	0.8	11	8,12	4,78	0,85
		Normalize	5.03	4.51	0.88	7	5.4	5.34	0.84	9	6.4	4,57	0,87
		Baseline	7.48	7.5	0.68	6	8.22	5.79	0.81	5	7.32	5,26	0,84
FTIR	7192	Raw	4,935	6,05	0,79	5	5.38	5.2	0,84	5	6.85	4.71	0,87
		Normalize	4,23	3,68	0,92	4	4.87	3.95	0.91	5	4.74	3.4	0,93
		Baseline	5.37	4.29	0.89	5	4.54	4.03	0.91	11	6.78	4.86	0,87
Low-level	7192	Normalize	3.27	3.3	0.94	6	3.83	3.36	0.94	5	3.9	3.16	0,95
		Baseline	5.2	4.57	0.88	5	4.17	3.87	0.91	12	6,54	4.94	0,84
Low-level with the selected features	1535	Normalize	4.34	4.22	0.9	7	4.96	4.44	0.89	9	7.2	4.34	0,89
		Baseline	6.52	5.18	0.85	7	6.23	5.76	0.81	11	10	7.34	0,69

Table S2. Statistical summary of the PLSR, PCR and SVR models for the prediction of SiO_2. The concentrations of SiO_2 in the analysed samples were in the range of 1.66–84.1 wt%.

Datasets/ Fusion Method	No. of Variables	Pre-Processing	PLSR				PCR				SVR		
			Calibration	Validation		Factors	Calibration	Validation		Factors	Calibration	Validation	
			RMSEcv	RMSEP	R^2		RMSEcv	RMSEP	R^2		RMSEcv	RMSEP	R^2
		Raw	8,94	7,95	0,87	4	5,98	8,22	0,86	7	12,9	10,3	0,74
MWIR		Normalize	7,84	7,77	0,88	3	8,02	8,8	0,84	4	7,98	8.47	0,86
	5520	Baseline	6,95	8,4	0,86	5	6,06	7,38	0,89	7	13.1	9.89	0,82
		Raw	12,42	12,8	0,67	4	8,07	9,69	0,81	5	10	9.13	0,83
LWIR		Normalize	5,52	6,12	0,92	4	5,3	6,5	0,91	5	6,33	6.555	0,9
	1672	Baseline	7,11	9,13	0,83	6	7,81	9,06	0,83	5	7.65	8.74	0,85
		Raw	7,97	6,95	0,9	4	5,52	7,55	0,88	9	11,88	9,14	0,86
FTIR	7192	Normalize	5,07	6,42	0,92	4	5,93	7,16	0,9	5	7,56	7.52	0,9
		Baseline	5,78	7,19	0,9	5	5,54	8,44	0,86	7	8,22	9.08	0,83
Low-level		Normalize	5,07	5,96	0,93	4	5,41	7,17	0,9	6	6,16	6.85	0,9
	7192	Baseline	6,44	7,66	0,88	5	7,22	8,56	0,85	6	8.4	8.69	0,89
Low-level with the selected features		Normalize	6,08	6,4	0,92	3	6,65	6,06	0,93	6	6.4	6,77	0,91
	3046	Baseline	7,02	8,3	0,86	7	7,98	8,37	0,86	9	10.77	10.1	0,81

Table S3. Statistical summary of the PLSR, PCR and SVR models for the prediction of Al_2O_3. The concentrations of Al_2O_3 in the analysed samples were in the range of 0.06–15.9 wt%.

Datasets/ Fusion Method	No. of Variables	Pre-Processing	PLSR				PCR				SVR		
			Calibration	Validation		Factors	Calibration	Validation		Factors	Calibration	Validation	
			RMSEcv	RMSEP	R^2		RMSEcv	RMSEP	R^2		RMSEcv	RMSEP	R^2
MWIR	5520	Raw	1,58	2,16	0,79	4	1,42	2,05	0,81	6	2.0	1.69	0,86
		Normalize	1,3	1,86	0,85	3	1,29	1,92	0,84	5	2.24	1.93	0,83
		Baseline	1,33	2,11	0,8	5	1,46	1,99	0,82	7	1.68	1.68	0,88
LWIR	1672	Raw	2,32	2,47	0,73	7	2,83	2,59	0,7	5	2.56	2.3	0,77
		Normalize	1,23	2.09	0,8	3	1,59	2,03	0,82	7	1.96	1.86	0,85
		Baseline	1,85	2,29	0.76	4	1,98	2,71	0.75	8	2.87	1.83	0,84
FTIR	7192	Raw	1,44	2,02	0,82	4	1,47	1,99	0,82	7	1,59	1.75	0,87
		Normalize	1,08	2,02	0,82	4	0,98	1,99	0,82	7	1.57	1.9	0,85
		Baseline	1,29	2,15	0,79	5	1,87	1,82	0,85	3	1.74	1.69	0,87
Low-level	7192	Normalize	1,35	1,95	0,83	4	0,93	2,06	0,81	9	1.97	1.83	0,84
		Baseline	1,37	2,06	0,81	4	1,3	2,13	0,8	10	1.7	1.68	0,88
Low-level with the selected features	2886	Normalize	1,79	1,4	0,91	6	2,1	1,48	0,9	9	2.12	1.79	0,86
		Baseline	1,82	1,82	0,85	6	1,66	1,77	0,86	9	2.00	1.59	0,89
